# The Role of ECG Strain Pattern in Prognosis after TAVI: A Sub-Analysis of the DIRECT Trial

**DOI:** 10.3390/life13061234

**Published:** 2023-05-24

**Authors:** Maria Drakopoulou, Georgios Oikonomou, Anastasios Apostolos, Maria Karmpalioti, Chryssa Simopoulou, Leonidas Koliastasis, George Latsios, Andreas Synetos, Georgios Benetos, George Trantalis, Skevos Sideris, Polychronis Dilaveris, Costas Tsioufis, Konstantinos Toutouzas

**Affiliations:** 1 First Cardiology Department, Hippokration Hospital, Athens Medical School, National and Kapodistrian University of Athens, 11527 Athens, Greece; mdrakopoulou@hotmail.com (M.D.); geooik88@gmail.com (G.O.); anastasisapostolos@gmail.com (A.A.); karmpaliotimaria@gmail.com (M.K.); synetos@yahoo.com (A.S.); benetosg@gmail.com (G.B.); geotrantalis@gmail.com (G.T.); hrodil1@yahoo.com (P.D.); ktsioufis@gmail.com (C.T.); 2 State Department of Cardiology, Hippokration General Hospital, 11256 Athens, Greece; skevos1@otenet.gr

**Keywords:** electrocardiographic strain pattern, transcatheter aortic valve implantation, self-expanding valve

## Abstract

Background: The presence of an electrocardiographic (ECG) strain pattern—among other ECG features—has been shown to be predictive of adverse cardiovascular outcomes in asymptomatic patients with aortic stenosis. However, data evaluating its impact on symptomatic patients undergoing TAVI are scarce. Therefore, we tried to investigate the prognostic impact of baseline ECG strain pattern on clinical outcomes after TAVI. Methods: A sub-group of patients of the randomized DIRECT (Pre-dilatation in Transcatheter Aortic Valve Implantation Trial) trial with severe aortic stenosis who underwent TAVI with a self-expanding valve in one single center were consecutively enrolled. Patients were categorized into two groups according to the presence of ECG strain. Left ventricular strain was defined as the presence of ≥1 mm convex ST-segment depression with asymmetrical T-wave inversion in leads V5 to V6 on the baseline 12-lead ECG. Patients were excluded if they had paced rhythm or left bundle branch block at baseline. Multivariate Cox proportional hazard regression models were generated to assess the impact on outcomes. The primary clinical endpoint was all-cause mortality at 1 year after TAVI. Results: Of the 119 patients screened, 5 patients were excluded due to left bundle branch block. Among the 114 included patients (mean age: 80.8 ± 7), 37 patients (32.5%) had strain pattern on pre-TAVI ECG, while 77 patients (67.5%) did not exhibit an ECG strain pattern. No differences in baseline characteristics were found between the two groups. At 1 year, seven patients reached the primary clinical endpoint, with patients in the strain group demonstrating significantly higher mortality in Kaplan–Meier plots compared to patients without left ventricular strain (five vs. two, log-rank *p* = 0.022). There was no difference between the strain and no strain group regarding the performance of pre-dilatation (21 vs. 33, chi-square *p* = 0.164). In the multivariate analysis, left ventricular strain was found to be an independent predictor of all-cause mortality after TAVI [Exp(B): 12.2, 95% Confidence Intervals (CI): 1.4–101.9]. Conclusion: Left ventricular ECG strain is an independent predictor of all-cause mortality after TAVI. Thus, baseline ECG characteristics may aid in risk-stratifying patients scheduled for TAVI.

## 1. Introduction

Over the last decade, transcatheter aortic valve implantation (TAVI) has been established as the primary treatment option for individuals with severe symptomatic aortic stenosis with increased surgical risk, and is steadily gaining traction as a valid alternative to conventional surgery in lower-surgical-risk patients [1,2,3]. The combination of considerable progress in transcatheter techniques and devices, along with the accumulating experience of operators have contributed to the minimization of periprocedural complications and have led to a dramatic improvement in TAVI short- and long-term outcomes [4,5,6,7,8,9]. Given the emerging expansion of indications for the minimally invasive, percutaneous repair of aortic stenosis, the challenge remains to identify those patients that are more likely to benefit from TAVI, as well as objective features that may be predictive of a less favorable prognosis.

Although the clinical value of new-onset conduction disturbances after TAVI has been previously discussed, evidence about the impact of baseline ECG characteristics on the prognosis of patients undergoing TAVI is still lacking [10,11]. An ECG strain pattern represents a well-recognized marker of left ventricular hypertrophy (LVH) and has been found to be associated with adverse clinical outcomes in aortic stenosis [12]. In the face of an increased afterload, LVH is considered as a physiological compensatory response to maintain cardiac performance in aortic stenosis, ultimately followed by progressive cell death and fibrosis, signaling the onset of symptoms and adverse cardiovascular events in the advanced stages of the disease [13]. Beyond its utility as a marker for LVH, ECG strain has been associated with increased myocardial injury and fibrosis, and has been proposed as a strong indicator of left ventricular decompensation in aortic stenosis [14]. Thus, there is considerable interest in identifying potential correlations between the presence of a baseline ECG strain pattern, as a marker of LVH and myocardial dysfunction, and the clinical outcomes after aortic valve replacement.

The DIRECT trial (Pre-dilatation in TAVI Trial) was a randomized clinical trial designed to evaluate the efficacy of TAVI with or without pre-dilatation in patients with symptomatic, severe aortic valve stenosis [15]. In this subgroup analysis of the DIRECT trial, we sought to investigate the prognostic impact of baseline ECG left ventricular strain on 1-year clinical outcomes after TAVI, including all-cause and cardiovascular mortality, stroke, heart failure hospitalization, and permanent pacemaker implantation.

## 2. Materials and Methods

### 2.1. Trial Design and Study Population

The main outcomes and the design of the DIRECT trial have already been published [15]. In short, consecutive patients with symptomatic, severe aortic valve stenosis undergoing TAVI with a self-expanding valve prosthesis between May 2015 and January 2018 at four centers in Greece and Israel were included.

The primary outcome of the trial was the difference in device success rates between TAVI performed with aortic valve balloon pre-dilatation (pre-BAV) and TAVI performed with no pre-dilatation (no-BAV) [16]. Severe aortic valve stenosis was defined as an aortic valve area of ≤1.0 cm^2^ (or aortic valve area index of ≤0.6 cm^2^/m^2^) by the continuity equation, and a mean gradient >40 mmHg or maximal aortic valve velocity >4.0 m/s by resting echocardiogram (or after dobutamine infusion if the subject had a stroke volume index below 35mL/m^2^ and a left ventricular ejection fraction below 50%). All subjects underwent physical examination, coronary angiography, echocardiography, and multislice computed tomography (MSCT) before the TAVI procedure. The transfemoral route was the preferred vascular access in all cases, except for those with unfavorable iliofemoral anatomy, where subclavian access was used. Transcatheter heart valve size selection was suggested to be performed according to the minimum measured annulus diameter by MSCT, in accordance with the established TAVI practice, but was finally left at the discretion of the physician. Patients with a bicuspid aortic valve were excluded. The study was registered under the ClinicalTrials.gov number NCT02448927.

In the present study, data from a sub-group of patients in the DIRECT trial, coming from one single center (Hippokration General Hospital of Athens) were analyzed. The study was conducted in accordance with the Declaration of Helsinki and it was approved by the Ethics Committee of the institution with the protocol number 23133, and date of approval being 23 December 2014. Informed consent was obtained from all subjects involved in the study. For each patient, the last ECG performed before TAVI was prospectively collected and retrospectively analyzed by two cardiologists in a blinded manner. Patients were divided into 2 groups: those with and those without an ECG strain pattern at baseline. Patients were excluded from the analysis if they had bundle branch block or a paced rhythm at baseline. All patients were prospectively followed up.

### 2.2. Electrocardiograms

All ECGs were recorded with a standardized protocol at 25 mm/s paper speed and 10 mm/mV amplitude. The last ECG performed before TAVI implantation was used for the analysis. An interpretation of the ECG was performed by two cardiologists who were unaware of the clinical and echocardiographic data. Typical left ventricular strain on ECG was defined as the presence of ≥1 mm convex ST-segment depression with asymmetrical T-wave inversion in leads V5 to V6 on a baseline ECG [17]. Other ECG features that were analyzed and recorded were the presence of an atrioventricular or an intraventricular block, and the duration of the PR and QRS intervals.

### 2.3. Study Endpoints

The primary endpoint of this study was the rate of death from any cause at 1 year after TAVI, defined according to criteria proposed by the Valve Academic Research Consortium-2 (VARC-2) [16].

Prespecified secondary endpoints included short-term (in-hospital, 30-day) mortality, rates of one-year cardiovascular mortality, rates of short-term and one-year stroke, rates of permanent pacemaker implantation after the procedure, and vascular complication rates.

As prespecified by the study protocol, all patients were followed up clinically and echocardiographically at 1 year after TAVI. At this time point, the incidence of the prespecified study endpoints and New York Heart Association (NYHA) functional class were recorded. In addition to this, a detailed echocardiographic examination was performed to assess myocardial function and bioprosthetic valve performance. Echocardiographic parameters included aortic valve effective orifice area, mean and peak transvalvular gradients, paravalvular regurgitation, left ventricular ejection fraction, and pulmonary artery systolic pressure. Structural valve deterioration was defined as valve-related dysfunction (a mean transaortic gradient ≥ 20 mmHg, effective orifice area ≤ 0.9–1.1 cm^2^, dimensionless valve index < 0.35, moderate or severe prosthetic valve regurgitation) or the need for a repeat procedure (SAVR or TAVR), according to VARC-2 criteria [16]. We arranged a follow up telephone call for the assessment of every patient that did not manage to attend the scheduled appointment.

### 2.4. Statistical Analysis

The analysis of data was conducted with the SPSS statistical package (version 26.0, IBM Corp., Armonk, NY, USA). Categorical variables are presented as percentages. Continuous variables presenting normal distribution are expressed as means ± one standard deviation (SD), while those non-normally distributed are expressed as median values (range). The Kolmogorov–Smirnov test was used to assess normality in the distribution of continuous variables. Comparisons between patients with baseline ECG strain and those without were conducted as follows: The chi-squared test or the Fisher’s exact test, when appropriate, were used for categorical variables. Continuous variables were compared using the Student’s *t* test, when normally distributed, or the Mann–Whitney U test. Potential correlations between different variables were evaluated with Pearson’s correlation or Spearman’s rank correlation test, as appropriate. Log-rank testing was used to evaluate differences in the event rates between the two study groups, and the respective Kaplan–Meier survival curves were created. A multivariable Cox proportional hazards regression analysis was performed by backward stepwise logistic regression to identify factors that were independently associated with the primary endpoint. Factors associated with the primary endpoint, with a *p* value below 0.2 in univariate analysis, were included in the multivariate model as potential confounders [18]. A two-tailed *p* value < 0.05 was considered statistically significant for all tests.

## 3. Results

### 3.1. Baseline Characteristics

During the study period, 171 patients undergoing TAVI in four centers were included in the registry of the DIRECT trial. Of these, 119 patients were recruited from one single center (Hippokration Hospital) and included in the present sub-analysis of the DIRECT trial. After the detection of left bundle branch block or paced rhythm at their baseline ECG before TAVI, five patients were excluded from the analysis.

In total, 114 patients were included in the final analysis. The baseline characteristics are presented in Table 1. The mean age of our cohort was 80.8 ± 7 years and 51 (44.7%) patients were females. The prevalence of arterial hypertension was similar between the two study groups. Balloon pre-dilatation was performed in 53 (46.5%) patients in the study cohort in contrast to 61 (53.5%) patients who underwent TAVI without pre-dilatation. A strain pattern on pre-TAVI ECG was present in 37 patients (32.5%).

At the time of the intervention, 27 patients were already carrying a permanent pacemaker. The rate of patients carrying a permanent pacemaker before TAVI was lower within the ECG strain group compared to those without ECG strain [4 pts versus 23 pts, 10.8 vs. 29.9 (% within groups), respectively]. Moreover, higher mean (54 ± 18 vs. 47 ± 12 mmHg, *p* = 0.018) and peak (90 ± 24 vs. 79 ± 20 mmHg) transaortic gradient values were recorded in patients with ECG strain in comparison to those without. Similar values of intraventricular septum and posterior wall thickness at end-diastole were recorded between the two study groups. However, there was a trend towards an increased left ventricular mass indexed (LVMI) in the ECG strain group, reflecting the higher degree of LV hypertrophy. No significant difference regarding left ventricular ejection fraction values (51.5 ± 8 vs. 50 ± 10%), or the mean EuroSCORE (24 ± 10 vs. 22 ± 8%) was observed. Regarding functional status, similar rates of patients with a NYHA score ≥ III (87 vs. 84.5%) were recorded in the two groups.

### 3.2. Clinical Outcomes

**Figure 1:**
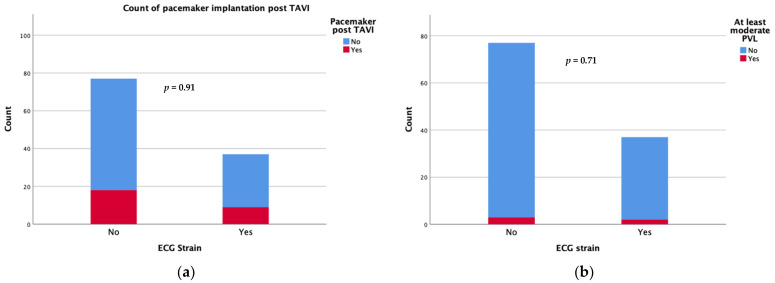
Count of pacemaker implantation (**a**) and moderate/severe paravalvular leak (**b**) at 30 days after TAVI.

Device success was achieved in 34 patients with an ECG strain pattern and in 67 patients without (91.9 vs. 87%, *p* = 0.335). Regarding the short-term complications following the TAVI procedure, there was no significant difference in new-onset conduction disturbances requiring permanent pacemaker implantation during the first 30 days after TAVI (24.3 vs. 23.4%) or the rate of moderate/severe paravalvular leak (5.4 vs. 3.9%) between patients with and those without ECG strain at baseline (Figure 1). There were also no differences in the rates of short term (30 days) major adverse events including strokes.

**Figure 2:**
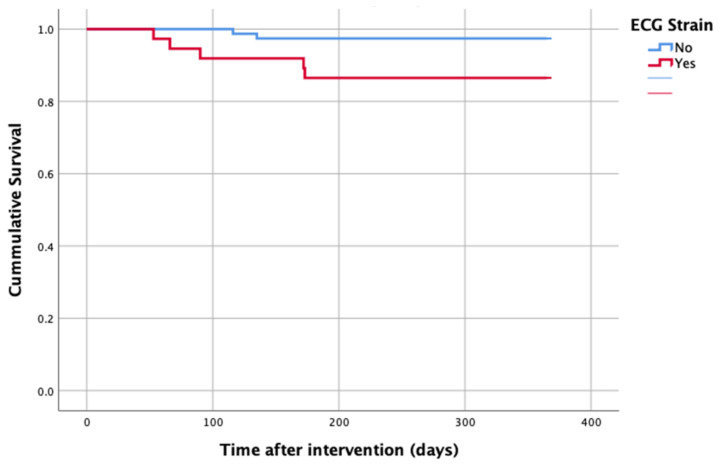
All-cause mortality—1 year after TAVI.

**Figure 3:**
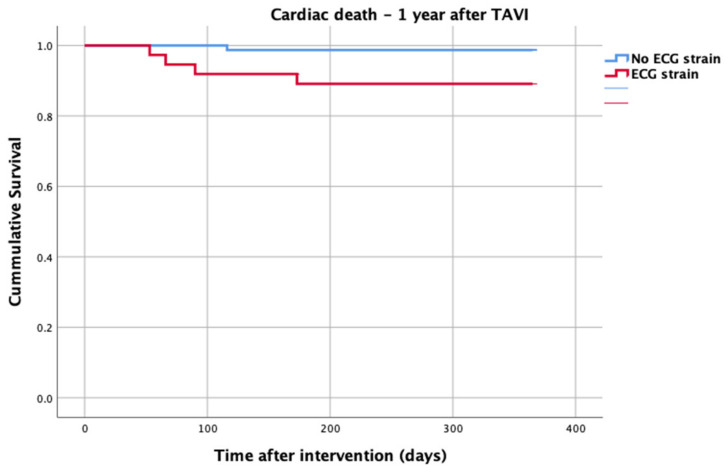
Cardiac death—1 year after TAVI.

**Figure 4:**
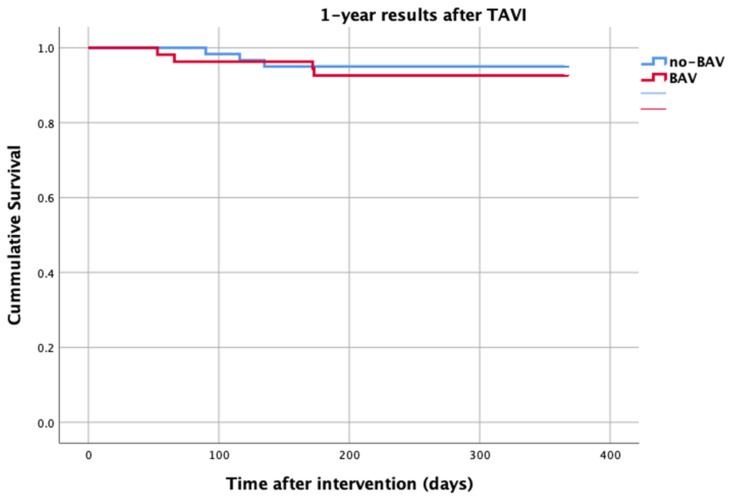
Stroke—1 year after TAVI.

At 1-year follow up, seven patients reached the primary clinical endpoint. More specifically, one patient died due to pulmonary infection, one patient due to infective endocarditis, one patient had a fatal pulmonary embolism, three patients died due to sudden cardiac death, and one patient died after acute heart failure decompensation. Patients in the strain group demonstrated significantly higher one-year all-cause mortality as well as cardiovascular mortality in Kaplan–Meier plots compared to patients without strain at baseline ECG (five vs. two, log-rank *p* = 0.02 and four vs. one, log rank *p* = 0.02) (Figure 2 and Figure 3). No difference regarding the stroke rates at the same period was observed (Figure 4). Within patients with an ECG strain pattern, two patients required hospitalization for heart failure decompensation compared to one in the non-ECG strain pattern group (5.4 vs. 1.3%, *p* = 0.2) (Table 2). The scheduled echocardiographic evaluation at 1 year after TAVI identified no difference in any echocardiographic parameters between the two study groups. Table 3 summarizes the echocardiographic variables at baseline, post-TAVI, and at 1 year. With regard to the effect of performing pre-dilatation before the implantation of the transcatheter valve in patients with baseline ECG strain, no difference was found between the two groups in regard to 1-year all-cause mortality (pre-BAV: four vs. no pre-BAV: three, log rank *p* = 0.164).

Along with the ECG strain pattern, aortic valve mean gradient at baseline (*p =* 0.056), the baseline EuroSCORE (*p =* 0.035), and the history of atrial fibrillation (*p =* 0.008) were inserted in a multivariate model which found that ECG strain is an independent predictor of all-cause mortality at 1 year after TAVI [Exp(B): 12.2, 95% Confidence Intervals (CI): 1.4–101.9, *p =* 0.02]. In the same multivariate model, the patient’s age, concomitant coronary artery disease, diabetes mellitus, and major periprocedural vascular events were assessed as potential confounders, but were not found to affect the significant association between ECG strain and all-cause mortality at 1 year after TAVI.

## 4. Discussion

The current study, a sub-analysis of the DIRECT trial on a cohort of 114 patients with severe symptomatic AS, aimed to specifically assess the prognostic impact of electrocardiographic strain pattern (ESP) on clinical outcomes after TAVI.

In our study, ESP was found in a significant proportion of patients with AS (32.5%). ESP was correlated with increased echocardiographic AS severity (mean gradient, peak gradient AVA, and AVAindexed) and increased all-cause mortality rates at 1-year follow-up. The performance of pre-dilatation of the native valve compared to a direct self-expanding valve implantation was not found to affect the predictive value of ECG strain regarding one-year TAVI prognosis.

In accordance with the results of our study, several reports have highlighted the frequent appearance and negative prognostic impact of ESP in patients with AS. The incidence of ESP in our population (32.50%) was similar to what was previously reported, ranging from 12% to 31%. Guinot et al. reported a similar rate of ESP (28%) among 390 patients with AS referred for isolated SAVR, albeit it consisted of a younger population (74 ± 10 years) with a lower risk profile (EuroSCORE II, 2.1% ± 1.5%).

It is becoming increasingly evident that aortic valve stenosis is not exclusively a disease of the aortic valve, but is also accompanied by secondary changes in the myocardium [19]. The increased afterload of the left ventricle induces a myocardial hypertrophic response that initially seems to compensate for elevated wall stress and maintain cardiac performance. However, there is a growing body of evidence supporting that a variation in the degree of LVH in patients with aortic stenosis exists, which, surprisingly, is not necessarily related to the severity of valve stenosis, but is rather modulated by other factors such as advanced age, male sex, obesity, genetic predisposition, and the presence of coexisting contributors [20,21]. Compared to moderate myocardial hypertrophy, an exaggerated hypertrophic response to comparable valve obstruction has been associated with increased mortality, more likely reflecting a link between the amplified hypertrophic process and premature progression to heart failure [22]. Histological data suggest that the transition from compensated hypertrophy to heart failure is dominated by increased myocyte damage and myocardial fibrosis, which constitutes an integral part of the hypertrophic process [23]. Notably, in patients with aortic valve stenosis treated either surgically or with TAVI, histological myocardial fibrosis was shown to independently predict impaired post-interventional recovery and poor survival [13,24]. ESP in patients with AS is highly likely to result from subendocardial ischemia. LV ESP is presumably the electrocardiographic epiphenomenon of chronic myocardial oxygen supply–demand mismatch, caused by altered coronary perfusion in the context of increased myocardial oxygen demand. The latter is attributed to the excessive LV mass (myocyte hypertrophy and addition of new myocytes to compensate for the increased wall stress and pressure overload) and the adverse LV remodeling in response to AS.

On this basis, reflecting an intensely hypertrophic myocardium, the ECG signs of left ventricular strain could contribute prognostic information on TAVI outcomes. This hypothesis is further encouraged by evidence supporting that ECG strain, apart from its well-known association with increased myocardial mass, represents a highly specific sign of myocardial injury and mid-wall myocardial fibrosis [14]. A more advanced stage in the trajectory of AS, which is that of LV fibrosis in response to increased pressure overload or myocardial ischemia, is likely to be reflected by ESP. Myocardial fibrosis is now a well-established irreversible marker of LV decompensation in AS, and is associated with poor long-term prognosis; yet, it is not routinely assessed in clinical practice. Cardiac magnetic resonance can comprehensively and non-invasively assess replacement fibrosis across the entire myocardium by means of late gadolinium enhancement (LGE), and the ongoing EVOLVED AS trial is trying to determine whether early valve intervention in asymptomatic patients with severe AS and midwall fibrosis can improve the adverse prognosis associated with midwall LGE. 

Indeed, the classic ECG strain pattern has gained considerable acknowledgment as an independent predictor of poor survival among patients with severe aortic valve stenosis awaiting valve replacement [14]. In asymptomatic patients with severe aortic stenosis, besides its ability to predict poor prognosis which was demonstrated in 1563 patients in the Simvastatin and Ezetimibe in Aortic Stenosis (SEAS) study, ECG strain was linked with a shorter asymptomatic period before decompensation and the onset of symptoms requiring valve replacement [12,25].

On the contrary, data regarding the prognostic impact of ECG strain on TAVI outcomes are still scarce. In line with our results, Al-Hijji et al. found that a pre-procedural ECG strain was an independent predictor of higher all-cause mortality at 3 years of follow-up [Hazard ratio 2.67, 95%CI (1.72–4.05); *p* < 0.001) among 290 patients with aortic stenosis undergoing TAVI [26]. In a cohort of 1122 TAVI candidates, ECG strain and bundle branch block identified patients with a more pronounced myocardial dysfunction, as defined by functional status and echocardiographic features, with a significantly higher incidence of major cardiovascular events occurring in the case of ECG strain or bundle branch block (HR 1.56, 95%CI 1.13–2.14, *p* = 0.006; HR 1.47, 95%CI 1.02–2.13, *p* = 0.04, respectively) during a mean follow-up of 4.5 years after TAVI [27]. Likewise, Guinot et al. reported a 4-fold increased risk of long-term mortality in surgically treated patients with ECG strain, which, unlike previously reported findings, was unrelated to the presence of preoperative left ventricular hypertrophy [28].

In the era of increasing TAVI implementation in younger and lower-surgical-risk patients, there is a pressing need to identify novel, alternative markers of myocardial dysfunction that could be concurrently utilized as powerful predictors of long-term clinical outcomes after the transcatheter treatment of aortic valve stenosis. In this regard, the interpretation of ECG characteristics emerges as a tempting field of research, given the low cost and rapid availability of the 12-lead ECG. Beyond ECG strain, advanced interatrial block at baseline, defined as a p-wave duration ≥120 ms with or without biphasic morphology in the inferior leads, has also been proposed as an independent predictor of all-cause mortality after TAVI [29]. Exceeding its value as a strong predictor for high-degree atrioventricular block requiring permanent pacemaker implantation, pre-existing right bundle branch block has also been linked to an elevated risk for sudden cardiac death during TAVI follow up [30].

Our study has several intrinsic limitations. Although performed by two independent cardiologists blinded to the patients’ baseline characteristics and procedural outcomes, the electrocardiograms were retrospectively analyzed which may constitute a bias for our study. While the primary target of this study was to assess the efficacy of pre-dilatation on TAVI outcomes, it was not designed to assess the prognostic value of ECG strain on TAVI outcomes, and, hence, the statistical power as well as the generalizability of our results may be limited.

## 5. Conclusions

In patients undergoing TAVI for severe symptomatic aortic valve stenosis, the presence of ECG strain is likely to represent a promising prognostic marker of long-term mortality, independent of LV hypertrophy. In view of its low cost and high availability, it may be important to prospectively investigate whether the presence of ECG strain should be used as an additional predictive factor for the evaluation of long-term clinical outcomes following TAVI.

## Figures and Tables

**Table 1:** Baseline clinical and echocardiographic characteristics of the study groups.

**Characteristic**	**ECG-Strain (*n* = 37)**	**No ECG-Strain** **(*n* = 77)**	***p* Value**
Age (years)	80.0 ± 7.2	80.7 ± 7.9	0.80
Male sex—*n* (%)	17 (45.9)	46 (59.7)	0.16
Body mass index (kg/m^2^)	27.4 ± 3.9	26.5 ± 3.2	0.19
Logistic Euroscore II	22.3 ± 8.1	24.0 ± 10.8	0.393
NYHA class III or IV—*n* (%)	35 (94.6)	73 (94.8)	0.87
Coronary artery disease—*n* (%)	19 (51.4)	34 (44.2)	0.471
Previous PCI—*n* (%)	5 (13.5)	4 (5.2)	0.123
Neurological disease—*n* (%)	2 (5.4)	5 (6.5)	0.82
Peripheral artery disease—*n* (%)	7 (18.9)	16 (20.8)	0.81
Diabetes mellitus—*n* (%)	11 (29.7)	21 (27.3)	0.78
COPD—*n* (%)	4 (10.8)	20 (26.0)	0.06
Hypertension	30 (81)	60(78)	0.942
Chronic kidney disease—*n* (%)	6 (16.2)	17 (22.1)	0.46
Atrial fibrillation—*n* (%)	14 (37.8)	30 (39.0)	0.90
Permanent pacemaker—*n* (%)	4 (10.8)	23 (29.9)	**0.025**
**Transthoracic echocardiography**			
IVS thickness at end diastole (mm)	12.2 ± 1.8	12.0 ± 1.9	0.53
PW thickness at end diastole (mm)	11.9 ± 1.4	11.5 ± 1.4	0.13
LVMI (g/m^2^)	118.7 ± 7.5	116.5 ± 8.2	0.09
Aortic valve velocity max (m/s)	4.70 ± 0.55	4.37 ± 0.56	**0.004**
AVA (cm^2^)	0.60 ± 0.13	0.68 ± 0.14	**0.004**
AVA indexed (cm^2^/m^2^)	0.32 ± 0.07	0.37 ± 0.07	**0.007**
Mean gradient-mmHg	54.3 ± 18.1	47.3 ± 12.1	**0.018**
Peak gradient (mmHg)	90.0 ± 24.6	79.0 ± 19.8	**0.015**
LV ejection fraction (%)	51.4 ± 7.8	50.1 ± 9.8	0.45
MR (moderate/severe)—*n* (%)	8 (21.6)	15 (19.5)	0.79
TR (moderate/severe)—*n* (%)	6 (16.2)	18 (23.4)	0.38
PASP (mmHg)	40 (35–100)	41.5 (30–95)	0.52

PCI: percutaneous coronary intervention; COPD: chronic obstructive pulmonary disease; IVS: interventricular septum; PW: posterior wall; AVA: aortic valve annulus; LV: left ventricular; MR: mitral regurgitation; TR: tricuspid regurgitation; PASP: pulmonary artery systolic pressure.

**Table 2:** Clinical endpoints at 30 days and 1 year.

	30-Days			1-Year		
Endpoint	ECG Strain *n* = 37	No ECG Strain *n* = 77	*p* (Log Rank)	ECG Strain *n* = 37	No ECG Strain *n* = 77	*p* (Log Rank)
Mortality—*n* (%)	0	0		5 (13.5)	2 (2.6)	**0.02**
Cardiovascular mortality—*n* (%)	0	0		4 (10.8)	1 (1.3)	**0.02**
Stroke—*n* (%)	0	0		1 (2.7)	0	0.147
Heart failure hospitalization—*n* (%)	0	0		2 (5.4)	1 (1.3)	0.2
New pacemaker implantation—*n* (%)	9 (24.3)	18 (23.4)	0.91	9 (24.3)	24 (31.2)	0.451

**Table 3:** Echocardiographic findings at baseline, post-TAVI, and at 1 year for both study groups.

	Baseline			Discharge			1 Year		
Characteristic	ECG Stain (*n* = 37)	No ECG Strain (*n* = 77)	*p*	ECG Strain (*n* = 37)	No ECG Strain (*n* = 77)	*p*	ECG Strain (*n* = 37)	No ECG Strain (*n* = 77)	*p*
AV Vmax (m/s)	4.70 ± 0.55	4.37 ± 0.56	**0.004**	1.9 ± 0.41	2.0 ± 0.46	0.10	1.7 ± 0.46	1.9 ± 0.49	0.10
Peak gradient (mmHg)	90.0 ± 24.6	79.0 ± 19.8	**0.015**	15.6 ± 6.4	18.2 ± 9.6	0.14	13.5 ± 7.0	15.6 ± 10.2	0.34
Mean gradient (mmHg)	54.3 ± 18.1	47.3 ± 12.1	**0.018**	8.4 ± 3.7	9.8 ± 4.9	0.13	6.9 ± 3.5	8.7 ± 5.4	0.14
AVA (cm^2^)	0.60 ± 0.13	0.68 ± 0.14	**0.004**	1.82 ± 0.36	1.84 ± 0.44	0.78	1.79 ± 0.25	1.79 ± 0.47	0.97
AVA indexed (cm^2^/m^2^)	0.32 ± 0.07	0.37 ± 0.07	**0.007**	0.99 ± 0.22	1.0 ± 0.25	0.86	0.98 ± 0.15	0.98 ± 0.24	0.90
PVL moderate/severe—*n* (%)				2 (5.4)	3 (3.9)	0.65	4 (10.8)	3 (3.9)	0.17
LV ejection fraction (%)	51.4 ± 7.8	50.1 ± 9.8	0.45	52.5 ± 7.9	51.5 ± 9.3	0.60	55.8 ± 7.1	52.4 ± 8.2	0.08
PASP (mmHg)	45.6 ± 14.0	47.4 ± 13.9	0.52	41.3 ± 15.4	41.4 ± 10.5	0.96	42.3 ± 13.2	39.0 ± 11.6	0.29

AV: aortic valve; AVA: aortic valve annulus; PVL: paravalvular leak; LV: left ventricular, PASP: pulmonary artery systolic pressure.

## Data Availability

The data presented in this study are available upon reasonable request.
